# Maternally Derived Antibody Levels Influence on Vaccine Protection against PCV2d Challenge

**DOI:** 10.3390/ani11082231

**Published:** 2021-07-29

**Authors:** István Kiss, Krisztina Szigeti, Zalán G. Homonnay, Vivien Tamás, Han Smits, Roman Krejci

**Affiliations:** 1 Ceva Phylaxia, Szállás u. 5., 1107 Budapest, Hungary; krisztina.richolm@ceva.com (K.S.); zalan.homonnay@ceva.com (Z.G.H.); 2 Functional Virology Research Team, Institute for Veterinary Medical Research, Hungária krt. 21, 1143 Budapest, Hungary; tamas.vivien@atk.hu; 3 Ceva Santé Animale, La Ballasteriere-BP 126, CEDEX, 33501 Libourne, France; han.smits@ceva.com (H.S.); roman.krejci@ceva.com (R.K.)

**Keywords:** PCV2, MDA, vaccination, protection

## Abstract

**Simple Summary:**

The efficacy of vaccination against type 2 porcine circoviruses is influenced by the level of maternally derived antibodies present at the time of vaccination of the piglets. This aspect was investigated by using two types of vaccines either comprised of a whole virus or its single protein. Both vaccines were able to overcome the impact of the maternal antibodies, however, the immunity, measured by serum antibody levels, developed faster for the whole virus type vaccine. The correlation between the maternally derived antibody levels at the time of vaccination and the challenge viral load showed differences amongst the tested lymph nodes. Additionally, it was confirmed also in this study that group oral fluid samples provide a reliable and relatively easy animal welfare-friendly way of estimating the PCV2 status of groups of pigs.

**Abstract:**

Piglets from a porcine circovirus type 2 (PCV2) stable farm of low and high levels of maternally derived antibodies (MDA) against PCV2 were vaccinated either with a whole virus type or a PCV2 ORF2 antigen-based commercial subunit vaccine at three weeks of age. Two non-vaccinated groups served as low and high MDA positive controls. At four weeks post vaccination, all piglets were challenged with a PCV2d-2 type virus strain and were checked for parameters related to vaccine protection over a four-week observation period. MDA levels evidently impacted the outcome of the PCV2d-2 challenge in non-vaccinated animals, while it did not have a significant effect on vaccine-induced protection levels. The humoral immune response developed faster in the whole virus vaccinates than in the subunit vaccinated pigs in the low MDA groups. Further, high MDA levels elicited a stronger negative effect on the vaccine-induced humoral immune response for the subunit vaccine than for the whole virus vaccine. The group-based oral fluid samples and the group mean viraemia and faecal shedding data correlated well, enabling this simple, and animal welfare-friendly sampling method for the evaluation of the PCV2 viral load status of these nursery piglets.

## 1. Introduction

Porcine circovirus type 2 (PCV2) and Porcine Circovirus Associated Diseases (PCVD, PCVAD) affect the pig industry worldwide, causing serious economic losses [1,2]. According to the current knowledge the virus consists of eight genotypes (PCV2a-PCV2h), of which PCV2d is the most prevalent genotype [3]. The covalently closed, circular, single-stranded DNA PCV2 genome consists of 1766–1768 nucleotides and is shaped by high-frequency mutations and recombination events [4,5]. Until recently, the available commercial vaccines have provided sufficient protection against the most prevalent genotypes of PCV2 [6,7,8].

Accordingly, controlling PCVD and the circulation of PCV2 is based on vaccination, supported inseparably by good hygiene and management practices. The efficacy of several commercial vaccines against either natural or experimental PCV2d infection, with the challenge system also used in this study, has already been previously demonstrated [7,9,10,11].

Vaccine efficacy is influenced by a number of factors, such as the age of the pigs and consequently, the level of maternally derived antibodies (MDA) and cell-mediated immunity (CMI) when vaccination or viral infection occurs, concomitant infection, and nutritional and environmental aspects, among others [9,12,13,14].

MDA may influence PCV2 vaccine protection, and the vaccination strategies must balance the potential advantage of delayed vaccination with obtaining the necessary immunity on time [15]. High levels of MDA were reported to interfere with the development of humoral immune responses upon PCV2 vaccination [16,17], although this interference was not significant [6,18], and even further, this interference in seroconversion did not consistently affect vaccine efficacy [19]. Nevertheless, protection against PCV2 infection and challenge significantly relies on CMI [10,20,21].

The objective of this trial was to investigate the potential effect of MDA on vaccine efficacy and compare two vaccines of different compositions, e.g., a whole virus and a subunit (capsid protein) product, against the experimental challenge infection by a widespread PCV2d-2 genotype isolate in piglets of a commercial source.

## 2. Materials and Methods

### 2.1. Allocation of Low/High MDA Groups

A farrow-to-finish farm of Large White + Duroc genetics with a lack of PCVD in the last two years, therefore considered a low “PCV2-pressure” farm, was selected as the source of the trial animals. The farm followed a protocol of vaccinating the piglets against PCV2 before weaning at 28 days of age. Two hundred piglets of 2.5 weeks of age from one and two parity sows were bled and tested by Ingezim Circo IgG 11.PCV.K.1. ELISA (INGENASA, Madrid, Spain), for the presence and levels of MDA against PCV2. Based on the results, low and high (L and H) MDA groups were established and divided into the different treatment groups (6 groups, 96 piglets altogether).

#### Group Assignments

Six groups were created, 16 pigs in each, comprising four vaccinated and two non-vaccinated groups with low and high (L and H) MDA counterparts per treatment type. The two vaccines used were CIRCOVAC^®^ (CV; Ceva-Phylaxia Ltd., Budapest, Hungary), an inactivated whole virus vaccine and Ingelvac CircoFLEX^®^ (CF; Boehringer Ingelheim Vetmedica GmbH, Germany), an ORF2 subunit vaccine and used according to the manufacturer’s instructions, at doses of 0.5 and 1.0 mL, respectively, applied intramuscularly (i.m.) at three weeks of age. The unvaccinated positive controls (PC) received 1.0 mL sterile PBS i.m. The resulting groups were then: HMDA/CV; LMDA/CV; HMDA/CF; LMDA/CF; HMDA/PC; and LMDA/PC.

### 2.2. Sample Collection

Blood samples and rectal swabs were collected before vaccination at 2.5 weeks of age, before challenge at 7 weeks, then weekly until slaughter at 11 weeks, for viraemia and shedding measurements. At the end of the study, mediastinal, mesenteric, and inguinal lymph nodes were collected for viral load quantification. After the challenge, oral fluid samples were collected from each group by letting the pigs chew on a cotton rope for 30 min. The squeezed oral fluid was then processed for qPCR (see below).

### 2.3. Challenge

Challenge was performed at four weeks post-vaccination (at seven weeks of age) by using the challenge virus D3276/5/16 PCV2d-2 strain [22] at a dose of 2 log9 genome copies/µL, 3 mL per nostril.

### 2.4. Serology

The collected serum samples were tested by Ingezim Circo IgG 11.PCV.K.1. indirect ELISA for IgG kinetics and by Ingezim Circovirus IgM/IgG 11.PCV.K.2. (INGENASA, Madrid, Spain) capture ELISA to measure IgM and IgG antibody positivity after challenge. The correlation between the increment (difference between the pre-vaccination and pre-challenge humoral immune response) and the MDA level measured prior to vaccination of the relevant groups was investigated and compared by linear regression. The rate of maternal PCV2 antibody decay for the PC groups was determined as previously described, based on the linear regression curve fitted to the logarithms of the ELISA titres [23,24].

### 2.5. qPCR

Viral DNA was extracted by the QIAamp DNA Mini Kit according to the manufacturer’s instructions. The quantitative real-time PCR was performed according to [25], using the following oligonucleotides: PCV2-84-1256U21 5′-gTA ACg ggA gTg gTA ggA gAA -3′; PCV2-84-1319L21 5′-gCC ACA gCC CTA ACC TAT gAC-3′; and TaqMan-1286-1314 FAM-ATg TAA ACT ACT CCT CCC gCC ATA CAA TC-BHQ1.

Virus suspension with known PCV2 titre and a reference plasmid containing PCV2 sequences was used to quantify the PCV2 virus load. For each reaction, the baseline and cycle threshold (Ct) number is determined automatically. The PCV2 copy number of the samples was calculated using dilution series (10^−2^–10^−8^) of a stock solution of a plasmid DNA (pGEM7+, Promega) containing the PCV2 whole virus sequence. Every 3.3 Ct difference equals with a 1 log difference in virus copy number.

### 2.6. Determination of Average Daily Weight Gain

The weight of the piglets was measured individually the day before vaccination, challenge, and at slaughter, and the group mean average daily weight gain (ADWG) was calculated between the vaccination and challenge and between the challenge and slaughter.

### 2.7. Statistical Analysis

A one-way ANOVA was used for comparing the means of the continuous data collected over time. Differences with *p* < 0.05 were considered significant. Linear regression was performed between the measured MDA at the day of vaccination and the titre increment before the challenge by using the Microsoft Excel 2016 program.

## 3. Results

### 3.1. Clinical Observations

The challenge infection did not induce any clinical alterations.

### 3.2. Serology

#### 3.2.1. MDA Measurements

**Figure 1:**
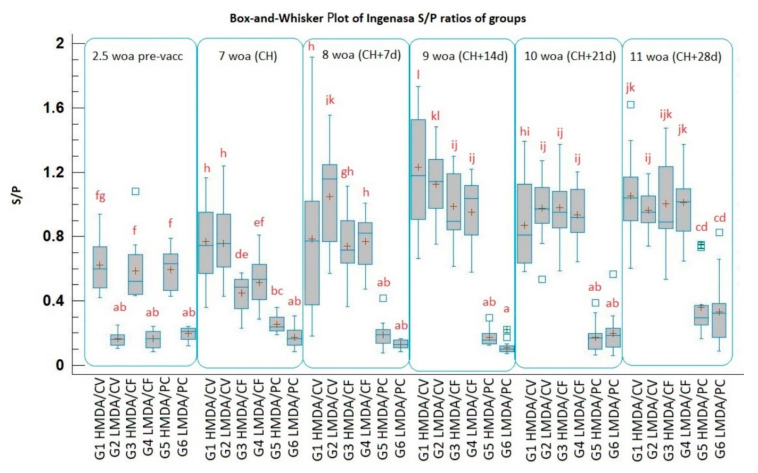
Box and whisker plots of sample-to-positive (S/P) ratios in the serum of pigs over time using the Ingezim Circo IgG 11.PCV.K.1. ELISA. S/P ratios above 0.2 are considered positive. A box represents the middle 50% of the data values. The continuous horizontal line within the box is the sample median. Plus indicates sample mean. Whiskers indicate the largest and smallest values. “Outside points” indicate values that are more than 1.5 times the interquartile range above or below the limits of the box. Different letter combinations indicate statistically significant group mean differences (*p* < 0.05). HMDA = high MDA; LMDA = low MDA; CV = Circovac; CF = Ingelvac CircoFLEX; PC = positive control.

The mean S/P ratio was 0.601 (0.43 and 1.08 as lowest and highest) for the H MDA groups and 0.174 (0.06 and 0.24 as lowest and highest) for the L MDA groups, equivalent with 434 (210 and 1692 as lowest and highest) and 92 (65 and 116 as lowest and highest) calculated titres, according to the manufacturer’s instructions of the ELISA test (Figure 1). The half-life of MDA for the H MDA PC group was calculated to be 45 days; however, for the L MDA PC group, with most of its members having antibody titres below the detection limit of the kit already from the start of the trial, this calculation did not make sense. The depletion of PCV2 MDA lasted until 8 weeks of age as demonstrated by the H MDA positive control group (in the L MDA positive control group it was low until the last sampling at necropsy), which was followed by an increase in IgG by 28 days post challenge (dpch) in both control groups, reaching the same levels (Figure 1).

#### 3.2.2. Post Vaccination Serology

By the time of the challenge all vaccinated groups had significantly higher antibody (Ab) titres than the non-vaccinated groups (*p* < 0.05). The titres of the vaccinates peaked at 14 dpch, the CV groups having significantly higher antibody levels than the CF groups.

The H and L MDA CV groups had equivalent Ab titres by the time of challenge, while the L MDA CF group had a higher mean titre than its H MDA counterpart.

The titre increase in the CF vaccinated groups reached a significantly lower level than the CV vaccinated groups until 14 dpch (*p* < 0.05), then persisted at this level until the end of the trial. The highest mean titre was measured for the H MDA CV at 14 dpch.

#### 3.2.3. Isotype Specific Post Challenge Antibody Responses

At CH there were 2/15 and 1/16 IgM positive pigs in the H and L MDA CV vaccinated groups, respectively, which decreased to 0 and 1 by 7 dpch. The rest of the animals were IgM negative at these two time points.

**Figure 2:**
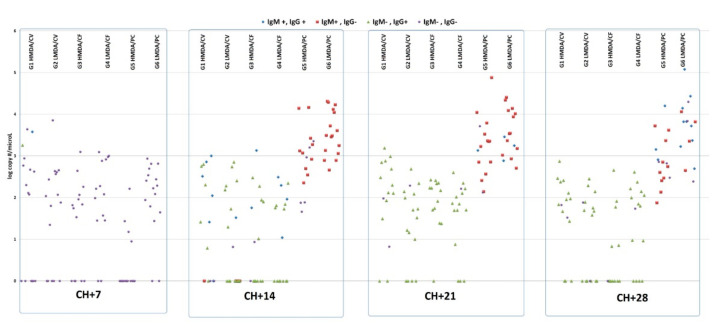
Scatterplot of the individual PCV2 amount (log copy per microliter) in serum and IgM/IgG positivity of pigs over time after the challenge. HMDA = high MDA; LMDA = low MDA; CV = Circovac; CF = Ingelvac CircoFLEX; PC = positive control.

**Figure 3:**
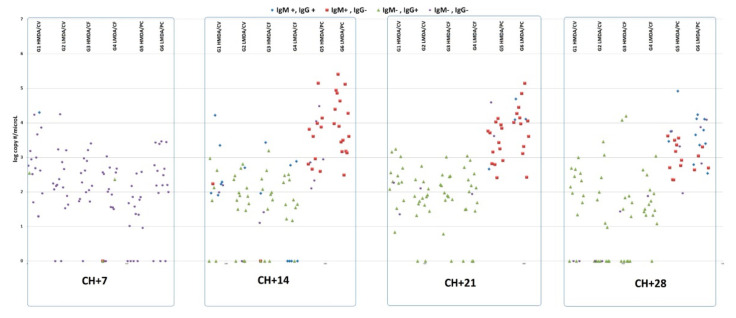
Scatterplot of individual PCV2 amounts (log copy per microliter) measured from rectal swabs and IgM/IgG positivity of pigs over time after challenge. HMDA = high MDA; LMDA = low MDA; CV = Circovac; CF = Ingelvac CircoFLEX; PC = positive control.

In the PC groups the first IgM positivity was detected at 14 dpch, reaching 100% and 63% positivity for the L and the H MDA group, respectively (Figure 2 and Figure 3).

All vaccinated groups had IgM-positive animals by 14 dpch, highest in the CV H MDA vaccinated group (44%, 7/15 pigs), but there were no further positives until the end of the study.

The first IgG positivity was detected already at 7 dpch, in the CV vaccinated groups. There was a clear difference between the IgG positivity ratio regarding the L and H MDA groups of both vaccines, e.g., the L MDA groups having higher percentages of IgG-positives through 14–28 dpch.

The L MDA vaccinated groups had peak IgG positivity at 14 dpch, while the H MDA vaccinated groups peaked a week later (according to the sampling schedule of the study), but by 28 dpch IgG positivity percentages decreased in all vaccinated groups.

In the PC groups the first detection of IgG positivity was at 21 dpch, the L MDA group demonstrating the higher percentage, and it rose further at 28 dpch, the end of the trial.

There was a negative correlation between the titre increment and the initial MDA level in both H MDA vaccinated groups, stronger in the case of the CF vaccinated one, having a correlation coefficient of −0.9065, and −0.6186 (*p* < 0.05), for the H MDA CF and the H MDA CV groups, respectively.

### 3.3. qPCR Results

#### 3.3.1. Viraemia and Challenge Virus Shedding

There was no viraemic pig at the time of challenge, while 4 animals (one in H MDA CV, one in H MDA CF, and two in L MDA PC group) shed PCV2 at such a low level (Ct 37.11–39.44, 35.0–8.2 calculated copy number equivalent) which prevented nucleotide sequence-based identification.

Challenge-induced viraemia was observed in all groups at 7 dpch, with the highest positivity ratio (87%) in the L MDA CF and in the non-vaccinated groups, and lowest (25%) in the H MDA PC group (Figure 2).

Starting from 14 dpch the non-vaccinated groups had 100% positivity for viraemia, which lasted throughout the trial. For the vaccinates this figure was uneven differing by one or two pigs regarding the positivity between the subsequent sampling occasions but consistently lower than the PC groups from 14 dpch onwards.

A similar pattern was observed for rectal shedding: from 14 dpch the PC groups showed 100% positivity until the end of the trial, while for the vaccinates this was uneven (Figure 3). Nevertheless, until 21 dpch the H MDA groups for both vaccines had slightly higher positivity ratios than the L MDA groups (100% vs. 88% and 92% vs. 87% for H and L CV and H and L CF, respectively), which continued until the last sampling for CV, but turned into the opposite for CF, i.e., the L MDA group having more positive animals (93%) than the H MDA group (62%).

Either for viraemia and virus shedding, the measured viral copy numbers showed significant differences between the PC groups and the vaccinates starting from 14 dpch (*p* < 0.05). The L MDA PC group had higher copy numbers at each sampling than its H MDA counterpart group, while the opposite was found for the CV vaccinated groups. Regarding CF, this difference between the two MDA level groups was inconsistent. The individual values, together with the IgM and IgG positivity are shown in Figure 2 and Figure 3.

#### 3.3.2. Oral Fluid Results

**Figure 4:**
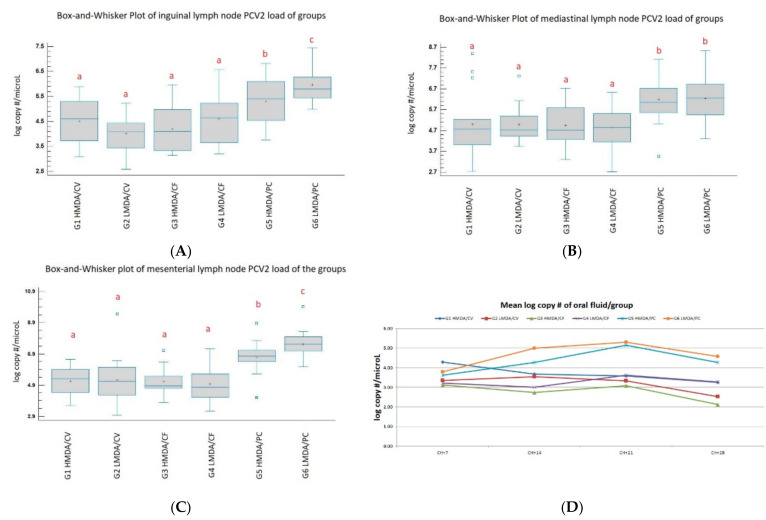
Viral load (log copy per microliter) of lymph nodes ((**A**): inguinal, (**B**): mediastinal, (**C**): mesenterial), and oral fluid (**D**) samples. Different letters indicate statistically significant group mean differences (*p* < 0.05). HMDA = high MDA; LMDA = low MDA; CV = Circovac; CF = Ingelvac CircoFLEX; PC = positive control.

Challenge virus excretion via the saliva was detected in all challenged groups from 7 dpch onwards throughout the trial period (Figure 4D). There was a positive correlation between the viral load measured in the group oral fluid and in the serum and rectal swab samples in the challenged groups (strongest in the PC animals). For the vaccinates the correlation coefficient was 0.7679 and 0.6769 for OF/rectal swab and OF/viraemia, respectively; for PC groups the correlation coefficient was 0.9069 and 0.8437 for OF/rectal swab and OF/viraemia, respectively.

#### 3.3.3. PCV2-Load of the Lymph Nodes

In general, the PC groups had a significantly higher PCV2 load than the vaccinated groups (Figure 4, values a–c). The H MDA PC group had a significantly lower PCV2 load than the L MDA group.

There were no differences (mesenteric and mediastinal lymph nodes) or only numeric differences (inguinal lymph nodes) between the viral loads of the vaccinated groups regardless of their initial MDA levels. The H MDA CV group had a numerically higher inguinal lymph node viral load than its L MDA counterpart group.

### 3.4. Average Daily Weight Gain (ADWG)

There was no significant ADWG difference among the groups between vaccination and challenge (data not shown).

**Figure 5:**
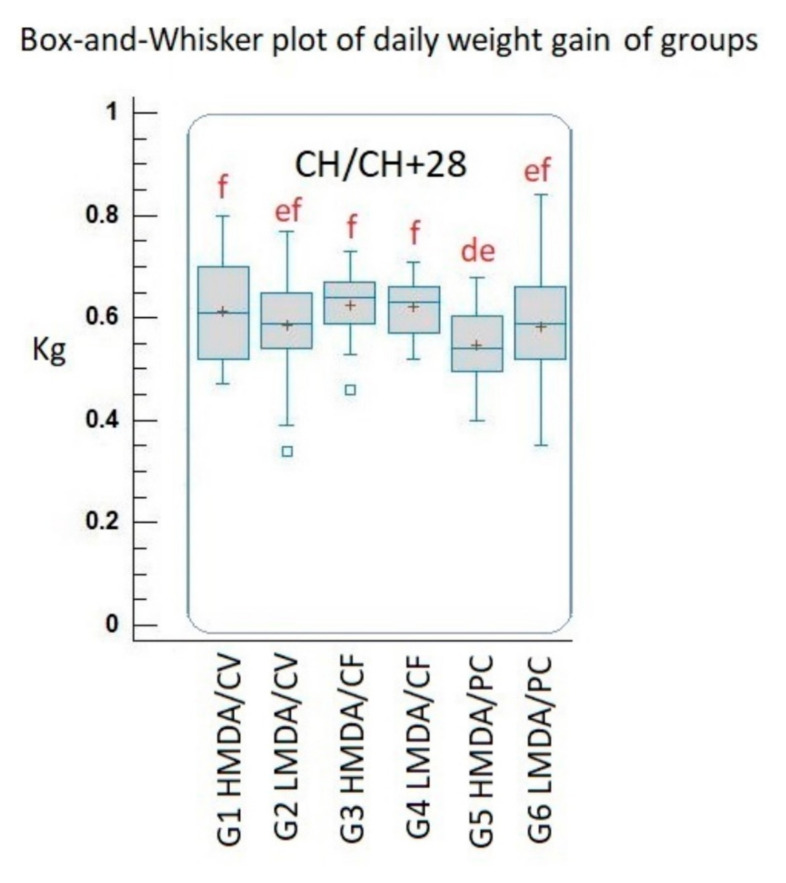
Box and whisker plots of the average daily weight gain data of groups. Different letter combinations indicate statistically significant group mean differences (*p* < 0.05). HMDA = high MDA; LMDA = low MDA; CV = Circovac; CF = Ingelvac CircoFLEX; PC = positive control.

Vaccination significantly improved the weight gain of the groups from challenge to the end trial, being unanimously better than the PC groups. Further, the H MDA PC group had a lower ADWG between the challenge and slaughter than the L MDA PC group, but the difference was not statistically significant (Figure 5).

## 4. Discussion

The effect of MDA on vaccine protection is a critical factor in the control of most infectious diseases. Reports concerning PCV2 vaccines and MDA influence provided important data from different trial/study settings, e.g., under field circumstances, using sow vaccination to ensure H MDA group of piglets, with a single or several vaccines in comparison, etc. [15,16,26]. It is fair to state that the effect of MDA cannot be predicted, and it depends on several factors [16].

This trial was performed with piglets that originated from a PCV2 stable farm, and without the induction of high MDA levels by sow immunization, and with the goal to compare two different types of PCV2 vaccines from the point of view of potential MDA interference. This selection of the source farm allowed a lower range of MDA titres than if sow vaccination had been applied (see Feng et al., 2016).

Maternally derived antibodies decay across a wide window of time (2–15 weeks of age; the mean antibody half-life was estimated to be 19 days in weanlings), depending on their initial concentration, and the lower it is the longer its half-life [23]. Our data supported this finding since we measured 45 days for the H MDA PC group, whereas in another similar trial, with a differing setup and method of serological analysis (i.e., immune-peroxidase monolayer assay), it was approximately 16 days for non-vaccinated piglets [19].

Challenge-induced IgG antibody titres were detected three and four weeks post challenge in the L MDA PC and H MDA PC groups, respectively, indicating some interference of MDA with the developing humoral immune response even at the time of challenge, when there was no statistically significant difference between the mean antibody titres of these two groups (Figure 1).

On the other hand, the difference between the ratio of the IgM positive animals at 14 dpch (16/16 vs. 10/16 for the L MDA PC and H MDA PC groups, respectively) indicated that the L MDA PC group was more susceptible to the challenge than its H MDA PC counterpart.

The vaccine-induced humoral immune response was evident by the time of challenge based on the significant antibody titre differences between the vaccinated and PC groups, in which differences peaked at 14 dpch, then decreased and showed a steady state between 21–28 dpch.

Although there were humoral immune response kinetic differences between the L and H MDA vaccinated groups, the L MDA animals responding faster to challenge than the H MDA ones. After 2–3 weeks this difference disappeared, indicating that vaccination was able to overcome the MDA variations, as was observed also by others [26,27].

It was demonstrated that MDA can have a negative impact on the antibody titre increment post vaccination, in an age-dependent manner: the earlier the vaccination, the higher the impact [15]. In this study, the linear regression indicated that there was a stronger negative correlation between the titre increment and the initial MDA level for the CF vaccinated group than for the CV vaccinated group. Nevertheless, it was proposed that maternally derived humoral immunity, rather than cell-mediated immunity interferes more with vaccine-dependent active humoral immunity [15]. The key role of cellular immunity, and, in particular, memory T cells, has been established in the immune responses against PCV2 [28,29]. Furthermore, the transfer of maternally derived cellular immunity and its protective effects was demonstrated by Oh et al., 2014.

There was a significant reduction of PCV2 viraemia and shedding for the vaccinates compared to the PC groups from 14 dpch onwards, which was in agreement with a previous report from a similar trial [8]. The increased ratio of viraemic pigs and the level of viraemia was linked with IgM positivity in the PC groups (Figure 2 and Figure 3), indicating the lack of pre-existing immunity against the virus challenge, while the vaccinates had a lower level of viraemia and shedding, and primarily IgG isotype antibody responses, as the consequence of the secondary antibody response.

The PCV2 loads in different lymph nodes had slightly different patterns: (i) altogether all lymph nodes in the vaccinated pigs had a lower amount of PCV2 DNA compared to the non-vaccinated groups; (ii) the viral load of the mesenteric lymph nodes correlated with the 2.5 weeks of age MDA levels in the PC groups; (iii) the viral load of the inguinal lymph nodes had numerically lower loads in the L MDA CV group than for the H MDA CV group.

The ADWG figures after virus challenge indicated a clear difference in favour of the vaccinated animals, however, such a short time interval would not allow relevant conclusions on this parameter.

The PCR results of the OF samples showed a good correlation with the group mean viraemia and shedding figures, rendering this sampling type as an animal welfare-friendly alternative to evaluate the PCV2 infection status of groups of pigs, in agreement with previous findings [30,31].

## 5. Conclusions

In this trial, the positive impact of the H MDA level was confirmed by protection against a challenge infection in PC animals. In vaccinated animals, the MDA level difference did influence the developing humoral immune responses, but vaccination was able to overcome this through the course of the study; though the whole virus vaccine induced a faster humoral immune response than the subunit vaccine. Both vaccines provided significant protection against the challenge with PCV2d-2 independent of the initial levels of MDAs, indicating that the vaccine-induced protection is less or not influenced by the level of MDA. The oral fluid sample can be recommended as a practical and feasible tool to estimate the PCV2 infection status of groups of pigs.
